# Paclitaxel and Therapeutic Drug Monitoring with Microsampling in Clinical Practice

**DOI:** 10.3390/ph17010063

**Published:** 2023-12-29

**Authors:** Mirjana Radovanovic, Peter Galettis, Alex Flynn, Jennifer H. Martin, Jennifer J. Schneider

**Affiliations:** 1 Centre for Drug Repurposing and Medicines Research, University of Newcastle, Callaghan, NSW 2308, Australia; peter.galettis@newcastle.edu.au (P.G.); alex.flynn@newcastle.edu.au (A.F.); jenniferh.martin@newcastle.edu.au (J.H.M.); jennifer.schneider@newcastle.edu.au (J.J.S.); 2 Drug Repurposing and Medicines Research Program, Hunter Medical Research Institute, New Lambton Heights, NSW 2305, Australia

**Keywords:** paclitaxel, therapeutic drug monitoring, Mitra^®^ microsampling, plasma, venous blood, capillary blood

## Abstract

Paclitaxel is an anticancer agent efficacious in various tumors. There is large interindividual variability in drug plasma concentrations resulting in a wide variability in observed toxicity in patients. Studies have shown the time the concentration of paclitaxel exceeds 0.05 µM is a predictive parameter of toxicity, making dose individualization potentially useful in reducing the adverse effects. To determine paclitaxel drug concentration, a venous blood sample collected 24 h following the end of infusion is required, often inconvenient for patients. Alternatively, using a microsampling device for self-sampling would facilitate paclitaxel monitoring regardless of the patient’s location. We investigated the feasibility of collecting venous and capillary samples (using a Mitra^®^ device) from cancer patients to determine the paclitaxel concentrations. The relationship between the venous plasma and whole blood and venous and capillary blood (on Mitra^®^) paclitaxel concentrations, defined by a Passing–Bablok regression, were 0.8433 and 0.8569, respectively. Demonstrating a clinically acceptable relationship between plasma and whole blood paclitaxel concentration would reduce the need to establish new target concentrations in whole blood. However, in this study, comparison of venous and capillary blood using Mitra^®^ for sampling displayed wide confidence intervals suggesting the results from the plasma and whole blood on this device may not be interchangeable.

## 1. Introduction

Since first marketed almost two decades ago, the taxane anticancer drug paclitaxel (PTX) has been used to treat many early and advanced solid tumors, namely, breast [1,2,3], ovarian [4,5], nonsmall cell lung cancer (NSCLC) [6], and head and neck carcinomas [7,8]. Taxanes produce their antitumor activity by binding to tubulin, stabilizing it, and preventing depolymerization, resulting in the inhibition of mitosis and cell proliferation and, ultimately, cell death [9].

While taxanes have substantial anticancer activity either alone or in combination with other antineoplastics, there is always a risk of adverse effects resulting in early cessation of therapy. The most common and life-threatening side effects observed with PTX are bone marrow suppression ((febrile) neutropenia, thrombocytopenia, and anemia) and colitis; however, other morbidities such as peripheral neuropathy, alopecia, hypersensitivity reactions, and fatigue are also common and debilitating [10].

PTX is administered by intravenous (IV) infusions usually lasting between 1 to 3 h, in weekly or 3-weekly cycles, as a monotherapy, but more often in combination with other antineoplastics such as platinum salts, cyclophosphamides, or monoclonal antibodies. Due to hypersensitivity reactions with taxanes, predominantly with the Cremophor^®^ EL formulation, premedication with corticosteroids and histamine receptor antagonists is often included in the treatment regimen.

Following administration, PTX undergoes substantial hepatic metabolism, biliary excretion, and fecal elimination [11]. Metabolism of PTX involves the hepatic cytochrome P450 enzymes, posing a risk for drug–drug interactions. Hepatic impairment can increase systemic exposure due to decreased drug clearance, placing patients who are not appropriately dose-reduced at an increased risk of toxicity [12]. Drug disposition is highly influenced both by protein binding [13] (>95% bound) and transporters [14]. The pharmaceutical formulation administered also affects the drug disposition [15]. PTX is also available as a targeted nanoparticle, albumin-bound formulation [16], which has the advantage of producing less systemic toxicity. PTX exhibits a high protein binding affinity, a large volume of distribution, and a nonlinear elimination rate due to saturable metabolism. When dosing as per manufacturer recommendations, large inter- and intraindividual variability in plasma drug concentrations achieved has been observed. The question concerning the utility of therapeutic drug monitoring (TDM) to reduce pharmacokinetic (PK) variability and adverse effects has been raised, given the reported relationships between systemic exposure and clinical response. Studies have shown that the time the concentration (Tc) of PTX exceeds 0.05 µM (~50 µg/L; Tc > 0.05) is a predictive parameter of hematological [17] and neurological [18,19] toxicity. The value for TDM of PTX was demonstrated by two large prospective phase 3 clinical trials [20,21]. Patients with advanced NSCLC were randomized to receive 3-weekly PTX and carboplatin with or without TDM. The target for PTX exposure was the duration of the plasma concentration above the threshold of 0.05 µM (Tc > 0.05 µM) for ≥26 and < 31 h. Treatment-associated toxicity, grade 4 neutropenia [21], and grade 2/3 peripheral neuropathy [20,21] were significantly improved in the TDM intervention groups. The final PTX doses were also significantly lower in the PK-guided groups, without a reduction in clinical effectiveness.

Joerger et al. [17] showed that patients with ovarian cancer treated with 3-weekly PTX and carboplatin had a better outcome in terms of disease progression when PTX Tc > 0.05 µM was >61 h. Similarly, Huizing et al. [10] showed an increase in patient survival with NSCLC when PTX Tc > 0.10 µM was >15 h. Two other clinical studies [22,23] also demonstrated the value of TDM of PTX. Overall, PK-guided dosing resulted in a significant reduction in PK variability and increased proportion of patients reaching the PTX PK targets (64% vs. 89%, [23]).

The evidence exists for the recommendation of PTX TDM, which can guide dose modification to increase efficacy (tumor progression and overall survival) [1,18,24] and prevent major toxicity such as severe neutropenia [17,25] and neuropathy [18,19]. The target PK parameter for PTX TDM can be determined by a single blood sample, obtained 24 h post infusion commencement, using a validated analytical method and readily available online software tools [25,26].

TDM requires a sample to be collected 24 h after the infusion commences, which is often inconvenient for patients/caregivers, particularly if they need to travel long distances to a clinic for blood collection. The collected venous blood samples must be centrifuged to obtain plasma for analysis upon arrival at the laboratory. Further, the conventional approach requires specialized personnel and infrastructure, often not available or limited in remote areas. Alternatively, enabling the patient/caregiver to self-sample by performing a fingerprick at home at the correct time and collecting a small drop of blood on a microsampling device such as the Mitra^®^, would remove barriers to TDM. Venous blood samples collected by phlebotomists may require refrigeration during transport to the analytical laboratory, if the drug is temperature sensitive, and centrifugation after arrival, while microsampling devices do not and may be sent to the laboratory via the normal mail system. We hypothesized that microsampling reduces the logistics requirements and cost factors and, most importantly, would facilitate TDM monitoring of PTX regardless of the patient’s location, providing there is a correlation between venous and capillary blood concentrations.

Given the potential advantages of microsampling, this study sought to assess the potential for using microsampling in the setting of PTX TDM. We aimed to investigate this by (1) investigating the relationship between plasma and whole blood PTX concentrations; (2) venous and capillary blood collected using Mitra^®^ microsampling devices; (3) conduct a pilot study to examine the feasibility of collecting a fingerprick sample, using a Mitra^®^ microsampling device, from cancer patients undergoing chemotherapy with PTX; and (4) perform a preliminary investigation of the relationship between venous plasma PTX concentration and the fingerprick (capillary blood) blood sample collected using a Mitra^®^ device. A new LC-MS/MS method was developed and validated for the purpose of undertaking this study.

## 2. Results

### 2.1. Method Validation

**Figure 1:**
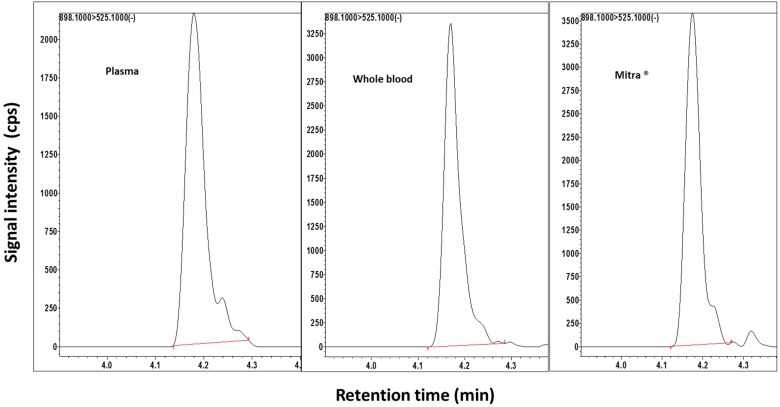
Chromatogram for paclitaxel in human plasma, whole blood, and Mitra^®^ microsampling devices at the lower limit of quantification (5 µg/L) (cps, counts per second).

PTX and the internal standard (IS) were eluted at 4.2 min under chromatographic conditions. A representative chromatogram for a lower limit of quantitation (LLOQ), 5 µg/L sample, in all matrices is shown in Figure 1. Mass spectra fragmentation data and the chemical structure of PTX are illustrated in the Supplementary Figure S1.

The assay was linear across the tested concentration ranges (5–5000 µg/L). The mean coefficient of determination (r^2^) was 0.9959 (SD = 0.0023); 0.9972 (SD = 0.0013) and 0.9983 (SD = 0.00011) for whole blood, Mitra^®^, and plasma assays, respectively. Patient samples with PTX concentrations > 5000 µg/L were diluted and reanalyzed. Precision and accuracy data are listed in Table 1.

The maximum interday imprecision was 11.0% for plasma and Mitra^®^ and 17.8% for whole blood matrix. Accuracy ranged from 91 to 113%. For the LLOQ, the coefficient of variation (CV) ranged from 5.7 to 19% and accuracy from 110 to 123%. No interferences were observed between the analyte and the IS, arising from the naturally occurring isotopes, nor were peaks observed in any of the matrices at the analyte retention time (R*t*) and multiple reaction monitoring (MRMs). Carryover in the first double blank was on average 38% of the LLOQ response, equivalent to a concentration of 1.9 µg/L; hence, reinjection of samples with low PTX concentration analyzed after a high concentration sample is required. PTX was stable in pure solution and in plasma and whole blood for at least 1 and 2 years, respectively, at −30 °C. The three freeze–thaw cycles did not affect the analyte stability in plasma. Shortterm stability for plasma matrix at room temperature (RT) and under refrigeration (4 °C) was proved for up to 24 h (quality control (QC) accuracy ranged 90–108%, n = 3 at four concentrations). Stability of PTX on Mitra^®^ devices was demonstrated for at least 15 months at RT (QC accuracy 80–95%, n = 3 at three concentrations) and for up to 3 days at 50 °C (QC accuracy = 87–101%, CV = 3–13%, n = 3 at four concentrations). Extracted samples were stable when sitting in the autosampler for 72 h. Matrix effect, recovery, and process efficiency were in the ranges 92–97%, 89–103%, and 87–95%, respectively, for plasma matrix and 94–104%, 52–67%, and 52–67%, respectively, for Mitra^®^ devices (n = 5 matrices, at two concentrations).

### 2.2. Patient Samples

**Figure 2:**
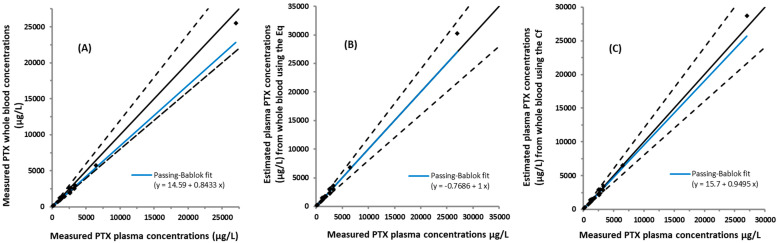
Passing–Bablok analysis of the venous plasma and whole blood paclitaxel (PTX) concentrations obtained from cancer patients (n = 21). The slope of a Passing–Bablok regression for the measured plasma and whole blood PTX concentrations was 0.8433 (95% confidence interval (CI) = 0.7608–0.9432) (**A**). Estimated plasma PTX concentrations from venous whole blood using a regression equation (Eq) from (**A**) (Blood = 14.59 + (0.8433 × plasma)) is represented in (**B**). Plasma estimated using a correction factor (Cf) (1.126) from the mean ratio (plasma/whole blood) is shown in (**C**). Slopes of Passing–Bablok lines for the graphs in (**B**,**C**) were 1.000 (95% CI = 0.9039–1.120) and 0.9495 (95% CI = 0.8533–1.061), respectively. Blue lines denote the Passing–Bablok fit, black lines the identity, and the black dotted lines the allowable range (±20% difference) from the identity line.

The relationship between measured plasma and whole blood PTX concentrations in samples obtained from cancer patients is represented by a Passing–Bablok regression (Figure 2A).

**Figure 3:**
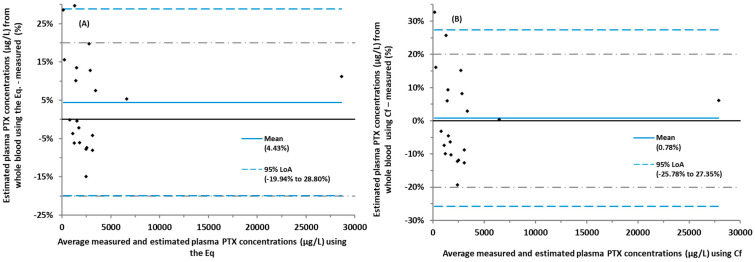
Bland–Altman bias plot of differences of the measured plasma paclitaxel (PTX) concentrations and estimated from whole blood (n = 21). (**A**) Measured plasma concentration vs. estimated using a regression equation (Eq) from Figure 2A (Blood = 14.59 + (0.8433 × plasma)). Mean difference (%) between the methods was 4.43% and the 95% of lower and upper limit of acceptance (LoA) was between −19.9% and 28.8%. (**B**) Measured plasma concentration vs. estimated using a correction factor (Cf) (1.126) from the mean ratio (plasma/whole blood). The mean difference (%) between the measured and estimated plasma PTX was 0.78%. The 95% LoA was between −25.78% and 27.35%. Gray dotted lines represent a 20% difference between the methods; blue dotted lines represent 95% LoA, while blue solid lines represent the mean.

The slope of a Passing–Bablok line was 0.8433 (95% confidence interval, CI = 0.7608–0.9432). The relationship between the measured vs. the estimated plasma PTX concentrations from whole blood using a (1) regression equation (Eq.) from the Passing–Bablok analysis in Figure 2A and (2) correction factor (Cf) based on the mean ratio of PTX plasma to whole blood concentrations are shown as a Passing–Bablok analysis (Figure 2B,C) and a Bland–Altman bias plot of differences (Figure 3A,B). The Cf based on the mean ratio of the measured plasma to whole blood concentrations was 1.126 (range = 0.810–1.367). Mean differences (%) between the measured plasma PTX concentrations and estimated from the whole blood represented by a Bland–Altman bias plot of differences (Figure 3A,B) were 4.43% and 0.78% for the Eq and the Cf methods, respectively. The 95% upper and lower limit of acceptance (95% LoA) for the Eq and Cf methods were −19.9% to 28.8% and −25.78% to 27.35%, respectively.

**Figure 4:**
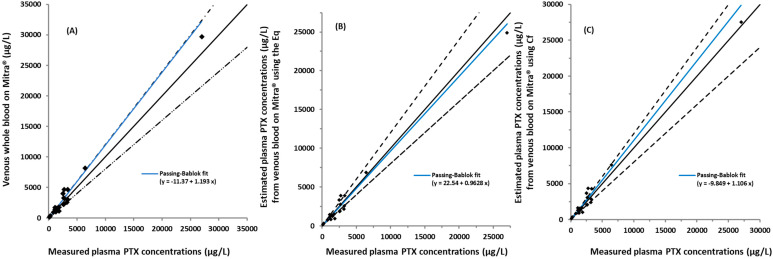
Passing–Bablok analysis of plasma and whole blood on Mitra^®^ paclitaxel (PTX) concentrations for venous blood obtained from cancer patients (n = 20). (**A**) Comparison analysis of the measured plasma PTX concentrations vs. venous whole blood on Mitra^®^. (**B**) Measured plasma vs. estimated plasma from venous whole blood on Mitra^®^ using the equation (Eq) and (**C**) using the correction factor (Cf) (0.928). Samples from twenty cancer patients were used for comparison. The slopes of the Passing–Bablok lines for the estimated concentrations using the Eq and the Cf were 0.963 (95% CI = 0.713–1.31) and 1.106 (95% CI = 0.868–1.521), respectively (**B**,**C**), while the slope for the measured plasma and whole blood on Mitra^®^ was 1.193 (CI = 0.930–1.61). Blue lines denote the Passing–Bablok fit, black lines the identity, and the black dotted lines the allowable range (±20% difference) from the identity line.

The relationship between measured PTX plasma and venous whole blood concentrations for Mitra^®^ devices was determined using a Passing–Bablok regression (Blood on Mitra^®^ = (1.193 × plasma) − 11.37), Figure 4A. Twenty sample pairs were used in the analysis, with one Mitra^®^ sample rejected due to incomplete filling of the device tip. The concentration range was between 180 and 29,687 µg/L in both matrices. Comparison of the measured plasma PTX concentrations vs. estimated using the Eq and the Cf (0.928) are shown in Figure 4B,C, respectively.

The Passing–Bablok regression equation from comparing the measured plasma PTX concentrations and Mitra^®^ fingerprick (capillary) blood samples was capillary blood on Mitra^®^ = (1.067 × plasma) − 412.8. Thirteen paired samples were used in the analysis with eight rejected due to incomplete sample collection by the Mitra^®^ devices. The slopes of the Passing–Bablok lines comparing the measured plasma concentrations to estimated using the Eq and the Cf were 0.963 (95% CI = 0.713–1.31) and 1.106 (95% CI = 0.868–1.521), respectively (Supplementary Figure S2).

To compare venous and capillary PTX concentrations using Mitra^®^ devices for sampling, twelve sample pairs were analyzed (nine samples rejected due to incomplete sampling) and a Passing–Bablok regression was used for data analysis. The slope of the regression line was 0.8569 (95% CI = 0.3924–1.096) (Supplementary Figure S3).

The recovery (%) of the estimated plasma concentrations from the whole blood and the venous and capillary blood using Mitra^®^ compared to the measured plasma is shown in Table 2. The estimated plasma from whole blood using the Eq and Cf resulted in 18 of 21 and 19 of 21 samples, respectively, within ±20% of the measured plasma concentrations. The plasma recovery (%) from venous and capillary blood using Mitra^®^ devices for sampling presented 10 of 20 and 5 of 13 samples, respectively, within ±20% of the measured plasma concentrations when using the Eq method (Table 2). Similar results were observed for the Cf approach.

## 3. Discussion

PTX TDM is currently performed by measuring the venous plasma PTX concentration. Demonstrating that a clinically acceptable relationship between plasma and blood PTX concentrations removes the need to establish a new target range when whole blood is used for analysis. In this study, PTX concentrations estimated from whole blood using the Eq from a Passing–Bablok regression and the Cf obtained from the mean ratio of plasma to whole blood were highly correlated to the measured plasma (Figure 2). Plasma estimation using both approaches, Eq and Cf, resulted in 18 of 21 and 19 of 21 samples, respectively, within ±20% of the measured plasma concentrations (Table 2).

The study also demonstrated that a venous PTX blood sample may be collected onto a Mitra^®^ device and extracted from the device for analysis. The measured plasma PTX concentration and estimated from the venous blood collected onto a Mitra^®^ device correlated well for both methods, (Eq and Cf), demonstrated by a Passing–Bablok regression analysis (Figure 4). This highlights a potential role for Mitra devices^®^ in simplifying sample transfer between a pathology collection center and the laboratory, removing the need to transport whole vials of blood under refrigerated conditions, thus reducing associated costs. Ideally, microsampling using fingerprick sampling and a device such as the Mitra^®^ would be performed by the patient in their own home. While most TDM for PTX is based on a single blood sample, this approach would also open the possibility for exploring the use of multiple samples and appropriate PK modeling approaches to further refine TDM. However, in this study, the preliminary data obtained comparing estimated plasma PTX concentrations using Mitra^®^ fingerprick samples does not yet support this application. The preliminary results indicate that while plasma PTX concentrations generally increased, the fingerprick Mitra^®^ values also increased (Figure S2). However, there was large variability between fingerprick Mitra^®^ and plasma PTX concentrations and fingerprick Mitra^®^ and venous whole blood Mitra^®^. The 95% CI for the slopes were wide, reflecting on the large differences observed between the measured and estimated PTX concentrations with 50% of the patients’ samples outside the ±20% agreement (Table 2). Since our study wanted to collect samples over a large range of concentrations, no set time for collecting the venous and fingerprick blood samples was specified, and samples needed to be collected while patients were attending the outpatient clinic. In this small number of samples collected, a large variability in the results was particularly evident at high concentrations. An additional factor to consider is the presence of higher concentrations of Cremophor^®^ EL in the first few hours during infusion, possibly affecting samples collected soon after the treatment commencement. Cremophor^®^ EL has been reported to influence the PK of PTX and the blood:plasma concentration ratio [27,28]. Whether this influences the distribution of PTX to capillary blood remains to be determined. A previous study using filter paper cards investigated using fingerpick blood samples collected on dry blood spot (DBS) cards and compared venous plasma PTX concentrations with DBS [29]. One study [29] collected samples from 34 patients and reported a correlation between plasma PTX concentrations and DBS in these patients, concluding that DBS represented a promising alternative. Samples were taken 18 to 30 h after a 1 h or 3 h PTX infusion; patient PTX concentrations measured in plasma and DBS ranged from approximately 10 to 250 µg/L, which were much lower than those observed in our study (130 to 30,000 µg/L). The DBS study used a correction factor for hematocrit effect since the cards do not collect a set volume, and this is a drawback to using card systems. Newer devices such as the Mitra^®^ used in our study collect a fixed volume of blood (10 µL), overcoming issues relating to hematocrit observed with filter paper card collection systems [30]. The preliminary data collected in our study are also rather encouraging to pursue volumetric Mitra^®^ fingerprick sampling further by studying samples taken at least 18 h after the infusion is ceased. Based on our observations, it would be worthwhile to collect 40–50 paired fingerprick samples [31] and venous blood samples from patients at times aligning with those used in the TDM studies.

Evidence concerning the usefulness of TDM for antineoplastic drugs such as PTX is emerging. Factors including other drugs coadministered with PTX, patient genetic factors, and comorbidities are all likely to contribute to PK variability. Exploring new approaches such as fingerprick blood sampling, which can be performed at home, should be undertaken to facilitate increased access to TDM for all patients. Increased access to TDM would benefit patients and health services by improving clinical outcomes and reducing toxicity.

## 4. Limitations

Although the clinic staff was shown how to collect fingerprick samples using Mitra^®^ devices, a significant number of samples received at our laboratory were not correctly collected. These samples were collected from patients who consented over a 2-year period. It is likely that several different nurses collected samples during this time and that some nurses may not have received sufficient training to ensure correct sample collection. This highlights the need for providing sufficient and ongoing training to ensure correct collection, which is highly relevant if, in the future, patients will be asked to collect samples at home.

As this was a pilot study, 21 patients were asked to participate. Owing to the loss of samples due to incorrect filling of devices, the comparison between fingerprick Mitra^®^ and plasma and fingerprick Mitra^®^ and whole blood venous Mitra^®^ only included 12 and 13 patients, respectively.

As discussed above, a limitation of this study was the ability to collect capillary and venous blood samples closer to the times which would be used in the clinical application of TDM. Acknowledging the relatively small number of patients in which comparison of fingerprick and plasma sample data were available, the results are encouraging and support continuing work to explore the implementation of dry blood sampling using a volumetric device such as the Mitra^®^ in the clinical and home setting.

## 5. Materials and Methods

### 5.1. Sample Collection

Twenty-one patients with cancer attending a chemotherapy outpatient treatment center and undergoing treatment with PTX, either alone or in combination with other antineoplastics, were recruited for this study. For inclusion in the study, patients had already undergone at least one cycle of chemotherapy or received oral therapy for at least one month and were willing to submit to a fingerprick plus venepuncture blood sample collection. Also, participants needed to be 18 years of age or older, have English proficiency, and be able to give informed consent. The nursing staff at the outpatient clinic were shown how to use the Mitra^®^ devices to collect fingerprick samples from patients. Patients underwent a venipuncture followed by a fingerprick blood collection on a Mitra^®^ device soon after the venous blood collection. The venous blood was collected into a K_3_-EDTA tube from which three types of samples were prepared: (1) an aliquot of the venous whole blood, (2) a venous Mitra^®^ whole blood prepared by dipping the tips into the tube, and (3) a plasma aliquot obtained after sample centrifugation. Plasma and whole blood aliquots were stored frozen (−30 °C) while Mitra^®^ samples were dried and kept in the clamshells at room temperature until analysis. Capillary whole blood samples were directly collected by using a lancet to prick the finger of the patient and produce a drop of blood. A sample (10 µL) was collected from this drop of blood onto a Mitra^®^ device. To collect during a broad range of times on the PK profile, samples were collected any time during the treatment or after treatment completion, with no emphasis placed on sample timing. Informed consent was obtained from patients prior to their participation. The study described was conducted in accordance with Declaration of Helsinki and local ethics approval was obtained prior to commencing the study (HREC/17/HNE/506).

### 5.2. Chemicals and Reagents

Paclitaxel (PTX) and paclitaxel ^2^H_5_ (IS) were purchased from Toronto Research Chemicals (PM Separation, Capalaba, QLD, Australia). LC-MS grade acetonitrile and methanol was from VWR (VWR, Tingalpa, QLD, Australia) and formic acid from Cova Chem, while water was prepared in-house using a Milli-Q (Q-POD^®^) Advantage A10 purification system (Merck, Macquarie Park, NSW, Australia). Drug-free human whole blood in K_3_-EDTA was obtained from healthy volunteers, while plasma was obtained from Australian Red Cross Services (Sydney, Australia). VAMS^TM^ Mitra^®^ microsampling devices (10 µL) were from Neoteryx (Torrance, CA, USA).

### 5.3. Equipment and Conditions

The liquid chromatography tandem mass spectrometry (LC-MS/MS) system was a Shimadzu ultrafast liquid chromatograph coupled with a triple quadrupole mass spectrometer (Shimadzu Oceania, Rydalmere, NSW, Australia). Compounds were chromatographically separated on a Kinetex C_18_ (50 × 2.1 mm, 1.7 µm) column via gradient elution using 0.1% formic acid in water and acetonitrile, as mobile phase A and B, respectively. The gradient starting at 20% B (0–2.3 min) was increased to 70% B (2.3–4 min), held at 70% B for half a minute (4–4.5 min) before returning to the initial conditions at 20% B (4.5–5 min). The flow rate was 0.5 mL/min, the column temperature was 40 °C, and the autosampler was maintained at 15 °C.

A tandem mass spectrometer, Shimadzu 8060, equipped with an electrospray ionization source interface operated in negative ion mode, was used for multiple reaction monitoring (MRM) analysis. The PTX and IS formic adducts ions in negative ionization mode were selected for monitoring due to higher signal intensity compared to the protonated ions observed in positive ion mode. The selected MRM for PTX were 898.10 → 525.10 (quantifier ion) and 898.10 → 121.00 → 319.05 (qualifier ions). For the IS, the MRM was 903.00 → 525.10 (quantifier ion) and 903.00 → 121.00 → 319.10 (qualifier ions). For data acquisition and processing, Shimadzu LabSolution software, version 5.96, was used. The optimized electrospray ionization source parameters were as follows: (1) nitrogen used as the nebulizing gas set to 2.0 L/min, which was also used as a heating and drying gas at a flow rate of 10 L/min; (2) capillary voltage applied to the electrospray ionization source probe was set to 3.5 kV; (3) for the collision-induced dissociation, high purity argon was used at a pressure of 270 kPa; and (4) the interface, heating block, and desolvation line temperatures were set to 300, 400, and 250 °C, respectively.

### 5.4. Preparation of Calibrators and Quality Control (QC) Samples

Stock solutions of PTX and internal standard (IS) were prepared at 1 mg/mL in methanol and stored at −30 °C. Intermediate calibrators and quality controls (QC) in methanol were prepared at ten times the concentrations of the working calibrators/QC. The intermediate calibrator concentrations were 0.05, 0.1, 0.5, 1, 2, 5, 10, and 50 mg/L while the QC samples were 0.08, 0.8, 4, and 40 mg/L. The working IS solution (200 µg/L) was prepared in acetonitrile. All solutions were stored at −30 °C in glass vials.

### 5.5. Preparation and Extraction Procedure for Plasma, Whole Blood, and VAMS^TM^ Samples for LC-MS/MS Analysis

For preparation of the working calibrators and QC samples, the intermediate calibrators/QCs, prepared in methanol, were diluted one in ten with, either drug free whole blood or plasma. The resulting calibrator concentrations were 5, 10, 50, 100, 200, 500, 1000, and 5000 µg/L. Working QC concentrations were 8, 80, 400, and 4000 µg/L. For calibrator 0, methanol was added to blank whole blood or plasma equivalent to the amount of methanol used to prepare the working calibrators. This was used to prepare a double blank sample, containing neither the analyte nor the internal standard, and a zero calibrator (blank whole blood/plasma with the internal standard). Working calibrators and QCs prepared in whole blood were also used to prepare the Mitra^®^ devices. The devices were dipped into the corresponding tubes until the entire tip turned red and dried in the clamshells, for at least 3 h, at room temperature (RT).

Samples were extracted using the following methods:

Plasma matrix: Plasma samples (50 µL) were precipitated with acetonitrile (100 µL) containing the internal standard (IS), centrifuged (5 min, 15,000× *g*), and 1 µL was injected into the LC-MS/MS.

Whole blood matrix: Whole blood (10 µL) was mixed with 10 µL of IS and 300 µL of acetonitrile:water (80/20 *v*/*v*) mixture. Tubes were sonicated (30 min), centrifuged (5 min, 15,000× *g*), and the supernatant was dried under vacuum (1 h, 40 °C). Samples were resuspended in an acetonitrile:water (20/80 *v*/*v*) mixture (20 µL), briefly mixed, and 1 µL was injected into the LC-MS/MS system.

Mitra^®^ samples: The tips were removed from the devices and placed into microfuge tubes to which 10 µL of the IS and 300 µL of acetonitrile:water (80/20 (*v*/*v*)) mixture were added. Samples were then treated the same way as the whole blood matrix except for the centrifugation step.

The Mitra^®^ microsampling device is equipped with a polymer tip attached to a plastic handle, which allows for collection of an exact volume of blood (10, 20, or 30 µL) from a fingerprick. The blood is absorbed into the tip, changing its color from white to red. The device is placed into the clamshell holder and allowed to dry at RT before extraction.

Mitra^®^ samples were inspected prior to analysis to ensure complete filling of the device. Samples showing presence of white areas on polymer tip were not included in the analyses. A schematic diagram of the PTX extraction protocol from plasma, whole blood, and Mitra^®^ is presented in Supplementary Figure S4.

### 5.6. Method Comparison and Statistical Analysis

The method comparison was performed using a Passing–Bablok regression analysis and a Bland–Altman bias plot of differences. The intercept of the Passing–Bablok is interpreted as the systematic bias (difference) between the two methods. The slope measures the amount of proportional bias (difference) between the two methods. Measured PTX concentrations in all three matrices (plasma, whole blood, and dry blood sampling) were compared using a Passing–Bablok analysis. Estimated plasma PTX concentrations were obtained from whole blood concentrations (venous and capillary) using (1) a correction factor determined from the mean ratio of the measured PTX concentrations in both matrices (plasma/venous whole blood, plasma/Mitra^®^) and (2) a regression equation obtained from a Passing–Bablok analysis of the measured concentrations. For calculating the percent (%) of estimated (recovered) plasma from whole blood matrices using the Passing–Bablok regression equation or the Cf, the estimated plasma PTX concentration for the corresponding whole blood matrix was divided by the measured plasma PTX concentration and multiplied by 100. Data were analyzed using Analyze-it^®^ software, version 5.68. For assessment of the agreement between the methods, a 20% difference from the mean for at least 67% of the results was set as the acceptance criteria [31].

### 5.7. Validation Protocol

The method was developed and validated for the purpose of undertaking this study. The validation was conducted in accordance with International Council for Harmonisation of Technical Requirements for Pharmaceuticals for Human Use (ICH) [32] guidelines in terms of linearity, accuracy and precision, specificity and selectivity, carry-over, and stability.

Eight nonzero calibrators, along with a double blank and a zero calibrator, were assayed to assess the linearity on three different days. Linear regression analysis, weighted 1/x using the peak area ratio (analyte/IS) versus the concentration, was used to determine the analyte concentration. Acceptance criteria for the interpolated values of each calibrator were set to ±15% from the weighed-in values, except the lower limit of quantitation (LLOQ) for which ±20% was allowed. Accuracy and precision were evaluated at four concentrations on three different days in triplicates. For specificity and selectivity, five whole blood/plasma matrices were extracted as outlined. The presence of the peaks at the analyte R*t* and their MRMs were visually assessed. This should be ≤20% of the response of the LLOQ and ≤5% of the IS response. Assessment of the cross-signal contribution between the analytes and the stable isotope IS was performed by individually injecting pure solutions of PTX and the IS into the LC-MS/MS. Peaks observed at the R*t* and MRM, except for the analyte being injected, were considered a cross-signal contribution. Acceptance criteria were set to the response being ≤20% of the analyte LLOQ and ≤5% of the IS. Matrix effect, recovery, and overall process efficiency for the three matrices was assessed at two concentrations. Carry-over was assessed by injecting a double blank extract after the highest calibrator. Response should not exceed 20% of the response of the LLOQ. Analyte stability in pure solution and plasma and whole blood at -30 °C was assessed for up to 1 and 2 years, respectively. Plasma stability was assessed at room temperature (RT) and refrigerated for up to 24 h. The freeze–thaw stability (−30 °C to ambient temperature) for plasma over three cycles was evaluated. Stability of the analytes on Mitra^®^ devices was evaluated at RT for up to 15 months and at 50 °C for up to 3 days. Stability of the extracted samples in the autosampler (15 °C) was evaluated for up to 72 h. Stability at the defined condition was accepted if the mean concentration was ±15% from the freshly prepared samples at the same concentration.

## 6. Conclusions

Using TDM in the clinical setting requires the availability of patient samples taken at an appropriate time and access to a validated assay technique. Access to sophisticated and expensive equipment such as LC-MS/MS required for PTX analysis may only be available at large metropolitan centers, limiting the opportunity for clinicians to use TDM routinely to personalize dosing, optimize outcomes, and reduce adverse effects. In recent years, microsampling techniques enabling patients to self-sample at home have been shown to be feasible for TDM of immunosuppressant drugs in the transplant setting [33]. Dry blood sampling also reduces the cost and logistics associated with transporting samples to the laboratory; some preliminary economic analysis in the transplant setting demonstrated cost-savings [34]. Given the advantages offered by TDM, it is important to explore how barriers to TDM can be overcome. This preliminary study has demonstrated that a venous blood sample could be collected on a Mitra^®^ device and sent to a central laboratory facility for analysis. Further, the agreement observed between plasma PTX concentration and the venous blood sample suggests easy conversion of the blood concentration to plasma concentration, enabling the use of the currently reported plasma target concentration. While it is acknowledged that the sample size in this preliminary study is small, the results indicate that dry blood sampling may be feasible for TDM of PTX, and further studies with a larger number of patients with sampling performed at 24 h are warranted. The study also highlights the importance of training in the proper collection of samples, an important lesson in the early stage of evolving new approaches to TDM.

## Figures and Tables

**Table 1:** Interbatch precision and accuracy of paclitaxel at four concentrations in plasma, whole blood, and Mitra^®^ samples.

		Plasma			Whole Blood			Mitra^®^	
Concentration (µg/L)	Mean Concentration ± SD (n = 13)	CV (%)	Accuracy (%)	Mean Concentration ± SD (n = 13)	CV (%)	Accuracy (%)	Mean Concentration ± SD (n = 13)	CV (%)	Accuracy (%)
5 ^#^	6.13 ± 0.36	5.8	123	5.9 ± 1.14	19	118	5.50 ± 0.32	5.7	110
8	8.22 ± 0.79	9.7	103	8.21 ± 1.47	17.8	103	7.96 ± 0.51	6.4	99.5
80	90.1 ± 6.5	7.2	113	84.5 ± 10.3	12.2	106	79.8 ± 7.3	9.2	99.7
400	420 ± 30	7.1	105	387 ± 69	17.8	97	412 ± 45	11.0	103
4000 *	4009 ± 353	8.8	100	3625 ± 640	17.7	91	4456 ± 389	9	111

SD, standard deviation; CV, coefficient of variation; Mitra^®^, a microsampling device. * n = 8; ^#^ n = 5.

**Table 2:** Estimated percent (E%) plasma recovery from whole blood and venous and capillary blood on Mitra^®^ devices using the equation (Eq) and the correction factor (Cf).

E % Plasma Recovery Using an Eq			E % Plasma Recovery Using a Cf		
Whole Blood	Whole Blood Mitra^®^	Fingerprick Mitra^®^	Whole Blood	Whole Blood Mitra^®^	Fingerprick Mitra^®^
111	101	119	106	111	120
114	143	93	109	158	105
122	133	147	116	147	175
135	105	n/A	130	115	n/A
96	69	60	92	76	64
94	51	76	90	56	73
114	n/a	84	110	n/A	74
94	66	147	91	72	156
93	74	n/A	88	81	n/A
86	72	71	82	80	74
112	92	n/A	106	102	n/A
100	80	n/A	96	88	n/A
100	98	100	97	107	69
133	122	n/A	139	127	n/A
93	104	n/A	89	115	n/A
98	86	n/A	94	95	n/A
117	131	287	118	141	176
92	80	92	88	88	106
96	130	124	93	143	120
106	106	n/A	100	118	n/A
108	117	141	103	129	172

## Data Availability

Data are contained within the article.

## Supplementary Materials

The following supporting information can be downloaded at: https://www.mdpi.com/1424-8247/17/1/63/s1. Figure S1. Mass fragmentation pattern of paclitaxel (PTX). Figure S2. Passing-Bablok analysis of plasma and capillary whole blood on Mitra® paclitaxel (PTX) concentrations from cancer patients (n = 13). Figure S3. Passing-Bablok analysis of venous and capillary whole blood paclitaxel concentrations collected using Mitra® microsampling devices from cancer patients. Figure S4. Schematic representation of paclitaxel (PTX) extraction from plasma, whole blood, and Mitra® microsampling devices.
