# Supplementary material for: Paclitaxel and Therapeutic Drug Monitoring with Microsampling in Clinical Practice
Source: Pharmaceuticals (Basel). 2023 Dec 29;17(1):63. doi: 10.3390/ph17010063 (PMC10820540; doi:10.3390/ph17010063)
Supplement: Supplementary file 1 [file pharmaceuticals-17-00063-s001.zip › pharmaceuticals-2743006-supplementary.pdf]

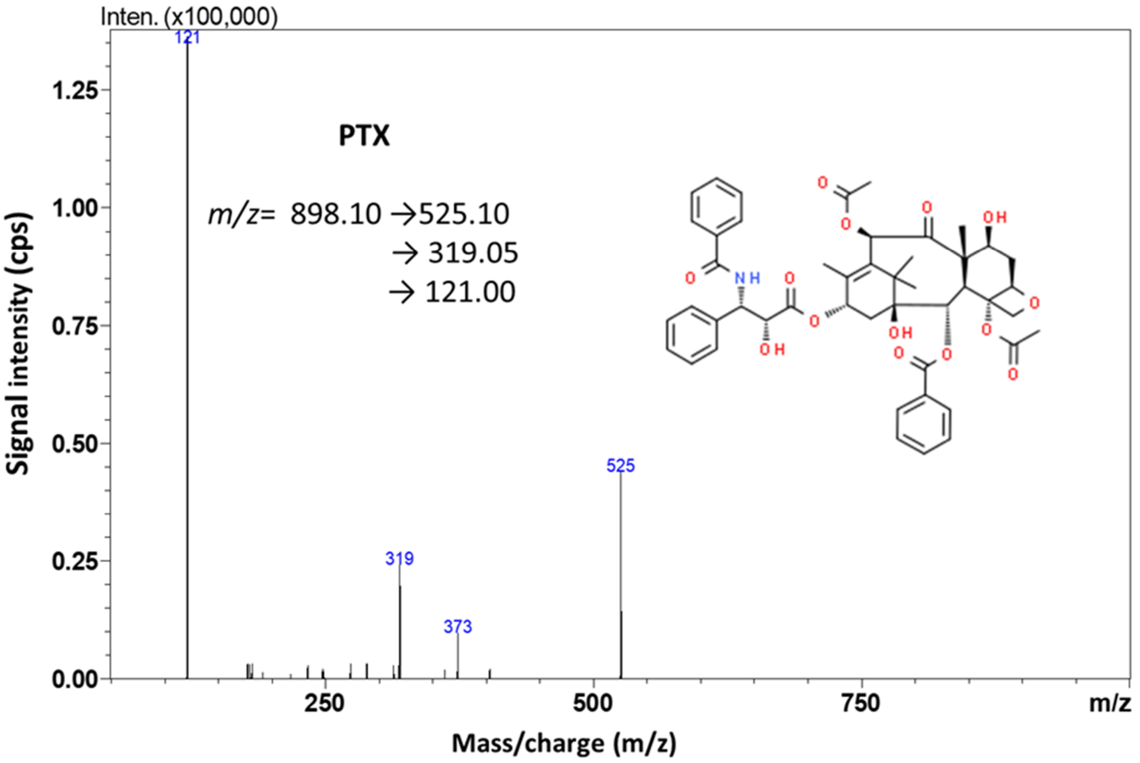

Figure S1. Mass fragmentation pattern of paclitaxel (PTX). The *m/z* ratio of 898.1 represents the formic adduct of paclitaxel precursor ion. The fragments *m/z* 525.1, 319.05 and 121.10 were selected for monitoring. PTX molecule chemical structure is shown on the right-hand side of the figure (molecular weight= 853.9 g/mol). *m/z*= mass to charge ratio; cps, counts per seconds

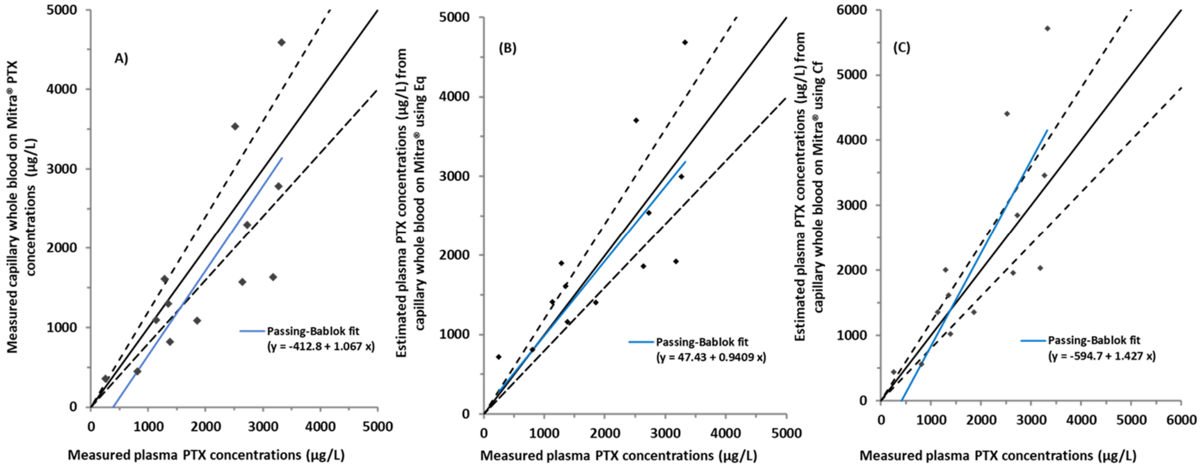

Figure S2. Passing-Bablok analysis of plasma and capillary whole blood on Mitra® paclitaxel (PTX) concentrations from cancer patients (n=13). A) Comparison analysis of the measured plasma PTX concentrations and capillary blood on Mitra® and B) measured plasma PTX *vs.* estimated plasma from capillary whole blood on Mitra® using the equation (Eq.) and C) using the correction factor (Cf) (1.245). The slopes of the regression lines for the Eq and Cf methods were 0.9409 (95 % CI=0.4792-1.658) and 1.427 (95 % CI=0.6366-2.317, respectively. Blue colored line denotes Passing-Bablok fit, black line is the identity, and the black dotted lines denote allowable range (± 20 % difference) from the identity line.

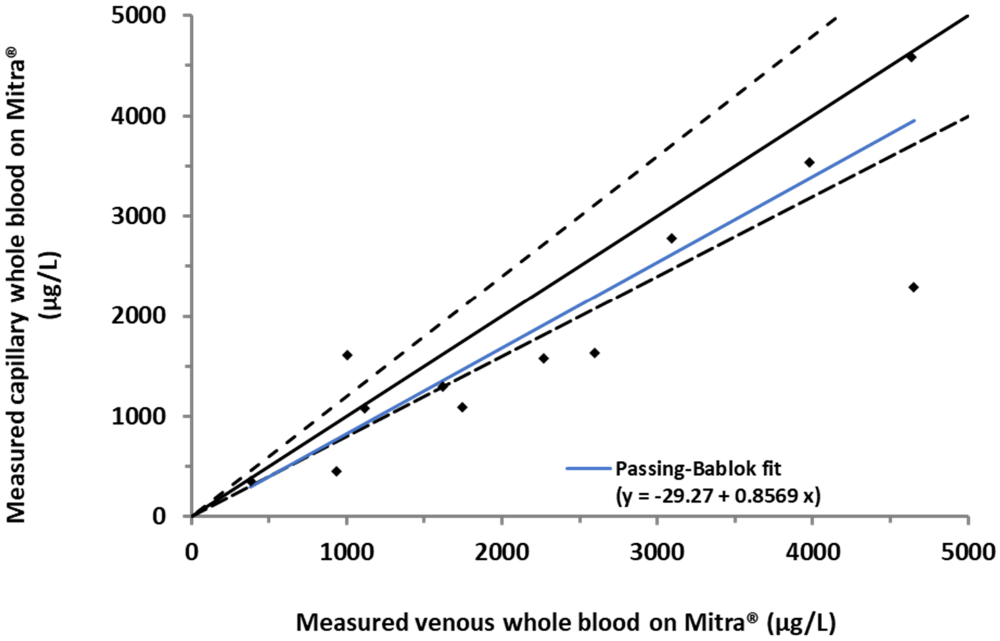

Figure S3. Passing-Bablok analysis of venous and capillary whole blood paclitaxel concentrations collected using Mitra® microsampling devices from cancer patients. Twelve sample pairs were analyzed with 9 rejected as inadequate. Slope of a Passing Bablok regression was 0.8569 (95 %CI= 0.3924-1.096). Blue colored line denotes Passing-Bablok fit, black line is the identity, and the black dotted lines denote allowable range (± 20 % difference) from the identity line.

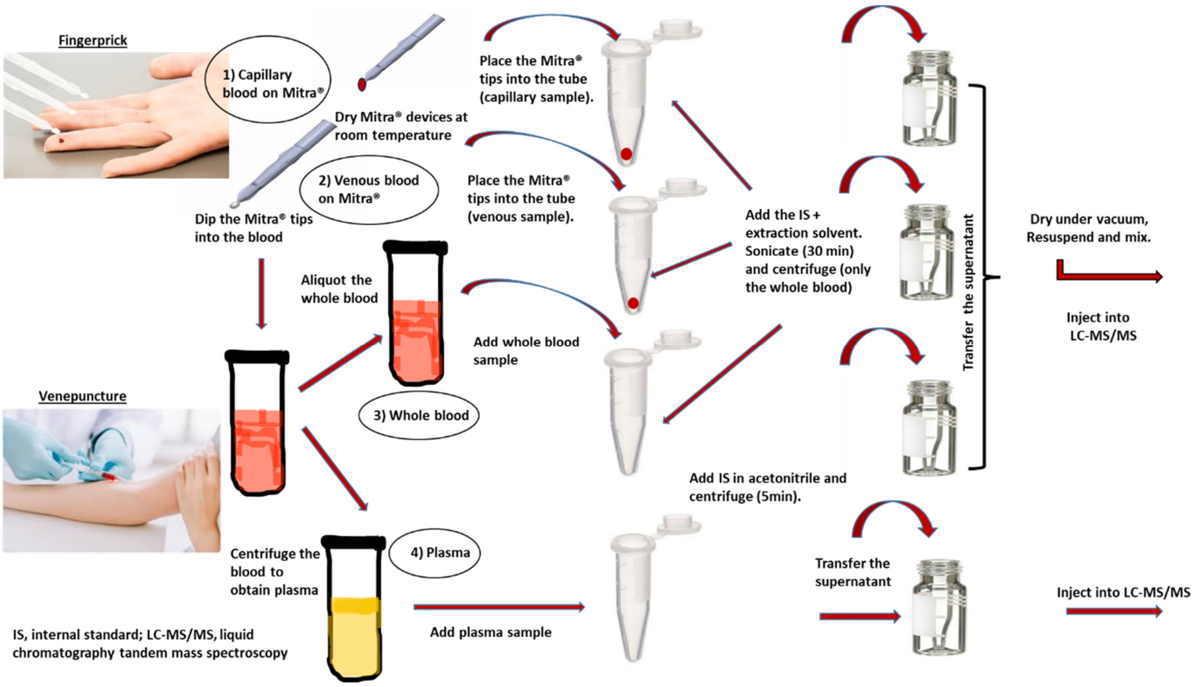

Figure S4. Schematic representation of paclitaxel (PTX) extraction from plasma, whole blood, and Mitra® microsampling devices.
